# Ocular Characteristics of Patients With Bardet–Biedl Syndrome Caused by Pathogenic BBS Gene Variation in a Chinese Cohort

**DOI:** 10.3389/fcell.2021.635216

**Published:** 2021-03-11

**Authors:** Xiaohong Meng, Yanling Long, Jiayun Ren, Gang Wang, Xin Yin, Shiying Li

**Affiliations:** ^1^Department of Ophthalmology, Southwest Hospital, Army Medical University (Third Military Medical University), Chongqing, China; ^2^Key Laboratory of Visual Damage and Regeneration and Restoration of Chongqing, Chongqing, China

**Keywords:** Bardet–Biedl syndrome, ocular characteristics, morphology, visual function, gene variation

## Abstract

Bardet–Biedl syndrome (BBS; OMIM 209900) is a rare genetic disease causing damage to multiple organs and affecting patients’ quality of life in late adolescence or early adulthood. In this study, the ocular characteristics including morphology and function, were analyzed in 12 BBS patients from 10 Chinese families by molecular diagnostics. A total of five known and twelve novel variants in four *BBS* genes (*BBS2*, 58.33%; *BBS4*, 8.33%; *BBS7*, 16.67%; and *BBS9*, 16.67%) were identified in 10 Chinese families with BBS. All patients had typical phenotypes of retinitis pigmentosa with unrecordable or severely damaged cone and rod responses on full-field flash electroretinography (ffERG). Most of the patients showed unremarkable reactions in pattern visual evoked potential (PVEP) and multifocal electroretinography (mfERG), while their flash visual evoked potentials (FVEP) indicated display residual visual function. Changes in the fundus morphology, including color fundus photography and autofluorescence (AF) imaging, were heterogeneous and not consistent with the patients’ functional tests. Overall, our study expands the variation spectrum of the *BBS* gene, showing that the ocular characteristics of BBS patients are clinically highly heterogeneous, and demonstrates the usefulness of a combination of the ffERG and FVEP assessments of visual function in the advanced stage of retinopathy in BBS.

## Introduction

Bardet–Biedl syndrome (BBS; OMIM 209900) is a genetic disease causing damage to multiple organs, including early onset progressive retinitis pigmentosa (RP), obesity, hypogonadal hypoplasia, hand and/or foot polydactyly, intellectual disability, abnormal renal development (Bardet, 1995; Biedl, 1995). Therefore, BBS patients’ lives can be seriously threatened. However, while the diagnosis of BBS is often missed, it is usually fully recognized when patients visit an ophthalmologic clinic due to impaired vision or night blindness.

The ocular symptoms of BBS mainly manifest as RP since childhood. Fundus manifestations include optic disk pallor, bone spicule pigment deposits, thinning of the blood vessels, and retinal osteocyte pigmentation (Riise et al., 1996; Moore et al., 2005). Moreover, scotopic rod and maximal responses as well as cone responses are non-detectable in most BBS patients (Berezovsky et al., 2012; Scheidecker et al., 2015). A total of 24 causative genes of BBS have thus far been discovered (Weihbrecht et al., 2017; Bölükbaşı et al., 2018; Mary et al., 2019; Wormser et al., 2019). Most of them encode proteins necessary for the formation of the BBSome multi-subunit complex which localized at the cilia and basal body, and their connecting the cilium in the outer segment of photoreceptor has been thought to play a role in protein trafficking (Wei et al., 2012; Xu et al., 2015). Defects in any one subunit will adversely affect the formation or function of the BBSome, causing the BBS phenotype (Khan et al., 2016; Priya et al., 2016; Weihbrecht et al., 2017). Moreover, rhodopsin in BBS gene mutant mice was incorrectly located in the cells of the inner segment (IS) and outer nuclear layer (ONL), and the OS, IS, and ONL exhibited progressive loss (Nishimura et al., 2004; Datta et al., 2015). Currently, BBS treatment focuses on managing diabetes, hypertension, and metabolic syndrome to minimize the secondary effects of the disease on vulnerable organs.

Most BBS cases reported worldwide are concentrated in Europe and the Middle East (Farag and Teebi, 1988, 1989), whereas the disorder is very rare among the Chinese population. In this study, we conducted clinical and genetic analyses of 10 Chinese families (12 patients) with BBS and comprehensively evaluated visual function and fundus changes in these patients. We also investigated BBS genotype-phenotype differences between Chinese reports and those from abroad.

## Materials and Methods

### Patient Recruitment

A total of 12 patients from 10 unrelated families with BBS treated at the Ophthalmology Department, Southwest Hospital, Army Medical University, Chongqing, China from 2012 to 2018 were retrospectively included in this study. The clinical diagnosis of BBS was made based on the potential patients presenting with four primary features or three primary features plus two secondary features. The primary features included retinal dystrophy, obesity, postaxial polydactyly, intellectual disability, gonadal abnormalities, and renal abnormalities. The secondary features included speech disorder/delay, sbtrabismus/cataracts/astigmatism, brachydactyly/syndactyly, developmental delay, polyuria/polydipsia (nephrogenic diabetes insipidus), ataxia/poor coordination/imbalance, mild spasticity (especially in the lower limbs), diabetes mellitus, dental crowding/hypodontia/small roots/high arched palate, left ventricular hypertrophy/congenital heart disease, hepatic fibrosis, short stature and hearing loss (Beales et al., 1999). If a patient was highly suspected to have BBS but one or two cardinal symptoms were missing, genetic analyses were performed for comprehensive analysis. We could clinically exclude the other syndromes such as Usher syndrome, Alström syndrome, Mckusick–Kaufman syndrome, Prader–Willi syndrome, and Joubert syndrome. Available family members of the probands were invited for clinical examination and genetic analysis. The research protocol was approved by the Ethics Review Board of Southwest Hospital (Chongqing, China, KY2020096), and the study was conducted in accordance with the provisions of the Declaration of Helsinki. Written informed consent was obtained from all participants. Assessment of ocular characteristics.

All patients underwent comprehensive ophthalmologic examinations, including best-corrected visual acuity (BCVA) tests with a decimal chart, indirect ophthalmoscopy, and slit lamp biomicroscopy. Color fundus photography, fundus fluorescein angiography (FFA), fundus autofluorescence (FAF; excitation: 488 nm), and Spectralis HRA-optical coherent tomography (OCT, Heidelberg Engineering, Dossenheim, Germany) imaging were used. Full-field flash electroretinography (ffERG; Diagnosys LLC, Lowell, MA, United States) and multifocal electroretinography (mfERG; VERIS Science 6.3.2; Electro-Diagnostic Imaging, Inc., Milpitas, CA, United States) were performed according to the ISCEV standard protocol (Hood et al., 2012; McCulloch et al., 2015). Pattern visual evoked potentials (PVEP) and flash visual evoked potentials (FVEP) were also recorded.

### Molecular Genetic Analysis

Genomic DNA was extracted from the patients’ peripheral blood using the Tiangen blood kit (Tiangen Biltech, Beijing, China) following the manufacturer’s standard sequencing protocols. Targeted next generation sequencing (NGS) was then performed using a capture panel including 131 known inherited retinal disease (IRD) genes (Supplementary Table 1). All coding exons of these genes were captured using the GenCap Liquid Phase Capture Kit (MyGenostics Inc.) and sequenced on the Illumina NextSeq 500. Then, the sequencing data processing, single nucleotide variants (SNVs) and insertions/deletions (InDels) calling and annotation were performed based on the hg19/GRCh37 human reference genome by using Haplotype Caller in GATK software (V.3.7), VariantFiltration in GATK software(V.3.7) and ANNOVAR software (V.3.4), respectively. Then, the online bioinformatics analysis software used to classify the pathogenicity of the candidate variants according to the American College of Medical Genetics and Genomics (ACMG) guidelines, including Exome Aggregation Consortium (ExAC), 1000Genomes database (1000G), and Genome Aggregation Database (gnomAD) to check the allele frequency; The rs number were found in Single Nucleotide Polymorphism Database (dbSNP); The pathogenicity of missense and synonymous variations were analyzed using ClinVar, HGMD Professional and four software prediction programs: Sorting Intolerant from Tolerance (SIFT), Protein Variation Effect Analyzer (PROVEAN) (Choi and Chan, 2015), Polymorphism Phenotyping v2 (PolyPhen2), and MutationTaster (Schwarz et al., 2014). The predicted effect on the splicing of all missense and synonymous variations was assessed with the Human Splicing finder program version 3.1 (Desmet et al., 2009). Finally, the disease-causing variants were identified while fully considering the clinical phenotypic findings and co-segregation analysis (by Sanger sequencing) of the affected subjects.

## Results

### Clinical Findings of BBS

**FIGURE 1:**
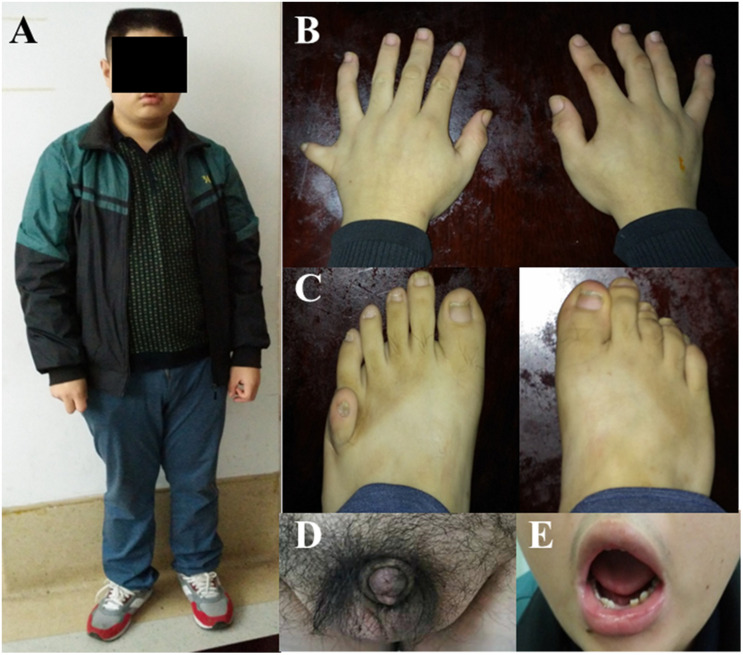
Systemic manifestations of BBS patients. The Wechsler Intelligence Score showed that this patient (F1-II:1) only had an 6-year-old intellectual level, his physical signs are: **(A)** Obesity (BMI 33.2 Kg/m^2^). **(B)** Polydactyly of the right hand and left foot. **(C)** Sexual organ dysplasia. **(D)** Dental dysplasia. **(E)** Abnormal tooth development.

We enrolled four women and eight men from 10 unrelated families. Their average age was 20.75 years (range: 8–37 years). The physical examination findings included obesity (12/12), hand and foot polydactyly (7/12), intellectual disability (4/12), abnormal tooth development (8/12), short stature (6/12), hearing loss (3/12), gonadal dysplasia (4/12), epilepsy (2/12), dorsal hemangioma (2/12), renal dysplasia (1/12), spinal dysplasia (1/12), gallstones (1/12), hyperlipidemia (1/12), hypertension (2/12), osteoarthritis-like changes (1/12), and heart abnormalities (1/12) (Table 1 and Figure 1). Five patients had undergone surgical extra toe removal at an early age, and one side ovary was removed from F6-II:1 due to acute torsion of a unilateral polycystic ovary cyst. Three patients dropped out of school at an early age due to intellectual disability.

**TABLE 1:** Systemic manifestations of patients with BBS.

											Abnormal						
	
Patient	Sex	Age at diagnostic exam	Initial eye symptom	Age of onset of NB	Ocular disease	BMI (Kg/m^2^)	Polydactyly	Intellectual disability	Gonad	Renal	Hear	Tooth	Short stature	Heart	Blood sugar	Blood pressure	Lipids
F1-II:1	M	21 y	NB	Since birth	RP	33.2	Yes	Yes	No	No	Yes	Yes	Yes	No	No	No	No
F2-II:1^#^	M	13 y	NB	Since birth	RP	19.53	No	Yes	No	No	No	Yes	No	No	No	No	No
F2-II:2	F	19 y	NB	Childhood	RP	24.44	Yes	No	OD	No	No	No	Yes	No	No	No	No
F3-II:1^#^	M	30 y	NB	Since birth	RP	26.72	No	No	EGD	No	Yes	No	No	No	No	No	No
F3-II:2*	M	29 y	NB	Since birth	RP	29.05	No	No	EGD	No	Yes	No	No	HSHR	No	SH	No
F4-II:1	F	37 y	NB	Since birth	RP	28.76	No	No	No	No	No	Yes	No	No	No	SH	No
F5-II:1	M	26 y	NB	Since birth	RP	32.30	Yes	No	–	–	–	Yes	Yes	–	–	–	–
F6-II:1	F	13 y	NB	3 y	RP	30.00	Yes	Yes	OC	–	–	Yes	Yes	–	SH	–	SH
F7-II:1	M	8 y	NB	Since birth	RP	26.30	–	No	–	–	–	Yes	Yes	–	–	–	–
F8-II:1	F	8 y	NB	8 y	RP	41.42	Yes	Yes	No	No	No	Yes	Yes	No	No	No	No
F9-II:1	M	27 y	NB	6 y	RP	26.12	Yes	No	No	No	No	Yes	No	No	No	No	No
F11F10-II:1	M	18 y	NB	Since birth	RP	30.42	Yes	No	No	RC	No	No	No	No	No	No	SH

EGD, external genital dysplasia; HSHR, high sinus heart rate; NB, night blindness; OC, ovarian cyst; OD, ovarian dysplasia; RC, renal cyst; SH, slightly higher.

*Bones and joints change. ^#^Epilepsy, dorsal hemangioma. Not available.

### Ocular Characteristics

**FIGURE 2:**
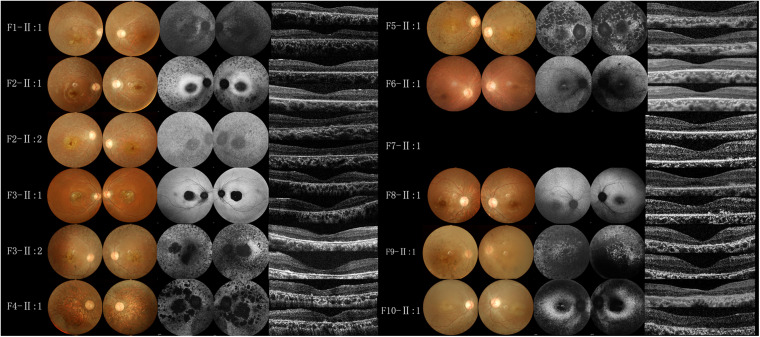
Fundus photography, fundus autofluorescence imaging, and foveal optical coherence tomography scans of probands from the BBS families. All patients had macular lesions, with pigmentary changes in the peripheral and midperipheral retina, attenuated arteriolar vessels, and pallor of the optic disk (Fundus). Plaque hypoautofluorescence in the macular area was enlarged in F1-II:1, F2-II:1, F2-II:2, F3-II:2, F4-II:1, F5-II:1, F9-II:1 (FAF). OCT (OD and OS in the vertical order) showed the outer structure of the subfovea was completely absent in F1-II:1, most of the outer structure remained in F6-II:1, and different degrees of deletion of the outer structure of the subfovea were observed in the rest of the patients (OCT).

**FIGURE 3:**
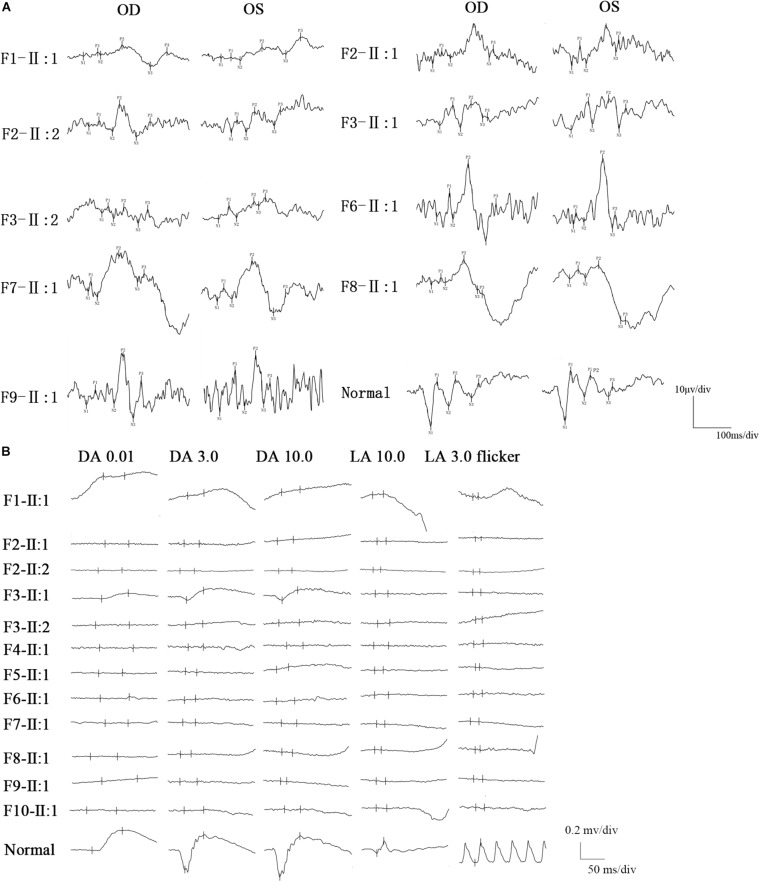
FVEP and ffERG recordings in patients with BBS. **(A)** The FVEP showed mild delayed but visible P2 waves in patients who performed it. Interestingly, all of six patients who showed moderate reduced P2 wave amplitude, also had BCVA better than 0.01 (F2-II:1, F2-II:2, F3-II:1, F6-II:1, F7-II:1, F8-II:1). **(B)** The ffERG was unrecordable in most of the patients, only patient (F3-II:1) had partial residual rod response.

All BBS patients were misdiagnosed with amblyopia upon their first visit to our hospital, and their common initial eye symptom was night blindness, and most of they have vision decline and vision field defect. Three patients (F-II:1, F2-II:2, and F8-II:1) had strabismus, and four (F1-II:1, F4-II:1, F5-II:1, and F8-II:1) had concomitant cataracts. F1-II:1 had nystagmus and concomitant cataract, and F2-II:2 had nystagmus with strabismus. The BCVA ranged from light perception (LP) to 0.3, including low vision (10/24 eyes) and legal blindness (16/24 eyes). All patients presented with typical RP features, including pigmentary changes in their peripheral and midperipheral retina, attenuated arteriolar vessels, pallor of the optic disk, and macular lesions (Figure 2; Fundus). Solitary macular atrophy was found in F3-II:1 and F8-II:1, which was characterized by pigment disorder, retinal thinning, and local retinal pigment epithelial atrophy, with a normal pigment change in the posterior pole retina. The AF of all patients showed plaque hypoautofluorescence in the posterior pole. Plaque hypoautofluorescence in the macular area was enlarged in F1-II:1, F2-II:1, F2-II:2, F3-II:2, F4-II:1, F5-II:1, and F9-II:1 (Figure 2; FAF). Patients’ fundus (F3-II:1 and F8-II:1) showed solitary enlarged macular lesions, while hypoautofluorescence of the macular area seemed to be normal in F10-II:1. OCT showed that the outer structure of the subfovea, including the myoid zone, ellipsoid zone, and external limiting membranes, was completely absent in F1-II:1; while most of the outer structure of the subfovea remained in F6-II:1; the rest of the patients showed different degrees of deletion of the outer structure of the subfovea (Figure 2; OCT). In the objective visual functional test, the ffERG displayed unrecordable waveforms in most of the patients (Figure 3A), and only F3-II:1 exhibited partial residual rod response. The mfERG waves were unrecordable in all patients assessed. The PVEP results showed a severely decreased amplitude and moderate delayed peak time in the P100 wave of F6-II:1, while the other patients had unrecordable PVEP due to nystagmus and fixation loss (Supplementary Figure 2). The FVEP showed mild delayed but visible P2 waves in patients assessed. Interestingly, all of the six patients who showed moderately reduced P2 wave amplitude, also had BCVA better than 0.01 (F2-II:1, F2-II:2, F3-II:1, F6-II:1, F7-II:1, and F8-II:1) (Figure 3B and Table 2).

**TABLE 2:** Fundus manifestation and evaluation of visual function in patients with BBS.

Patient	Ocular complications	BCVA RE/LE	Fundus	PVEP (P100 wave)	FVEP (P2 wave)	ffERG	mfERG
F1-II1	Nystagmus, cataract	CF/HM	MA, ODP, OCLPD	UR	Severe reduce amplitude, mild delay	UR	UR
F2-II:1	Strabism	0.05/0.2	MA, ODP, OCLPD	Severe decreased amplitude and moderate delay peak time	Moderate reduced amplitude, mild delay	UR	UR
F2-II:2	Pystagmus, strabism	0.05/0.1	MA, ODP, OCLPD	UR	Moderate reduced amplitude, mild delay	UR	UR
F3-II:1	–	0.02/0.01	MA, ODP, OCLPD	UR	Moderate reduced amplitude, mild delay	Partial residual rod response	UR
F3-II:2	–	LP/LP	MA, ODP, OCLPD	UR	Severe reduced amplitude, without delay	UR	UR
F4-II:1	Cataract	HM/HM	CS, RPE atrophy, ODP	–	–	UR	UR
F5-II:1	Cataract	0.05/0.05	MA, ODP, OCLPD	UR	–	UR	UR
F6-II:1	–	0.1/0.15	ODP, OCLPD	Moderately decreased amplitude and delayed peak time	Moderate reduce amplitude, mild delay	UR	–
F7-II:1	–	0.2/0.2	MA, ODP, OCLPD	–	Moderate reduceamplitude, mild delay	UR	–
F8-II:1	Exotropia	0.3/0.3	RPD	–	Moderate reduceamplitude, mild delay	UR	–
F9-II:1	Cataract	HM/HM	ODP, OCLPD,	–	Unstable and severe reduced amplitude, mild delay	UR	UR
F10-II	–	0.2/0.1	MA, ODP, OCLPD	–	–	UR	UR

*BCVA, best-corrected visual acuity; CF, count finger; CS, choroid sclerosis; HM, hand move; LE, left eye; LP, light perception; MA, macular atrophy; RE, right eye; OCLPD, osteocyte cell-like pigment deposition; ODP, optic disk pallor; RPD, retinitis pigment disorder; UR, unrecorded; –, not available.*

### Variant and Pedigree Analysis

**FIGURE 4:**
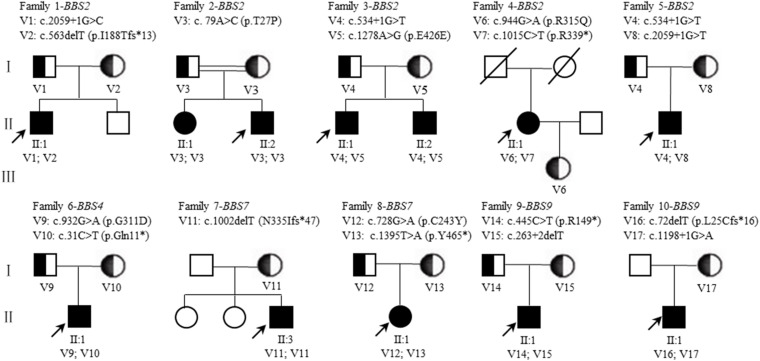
Pedigrees of BBS families. The squares and circles indicate men and women, respectively. The black solid square or circle represent the affected individual. The half black solid circle or square represent variation carrier. The probands are indicated by arrows. V, variant. A double line indicates a consanguineous marriage (The parents of F2-II:1 and F2-II:2 are consanguineous). A diagonal indicates that the individual has died.

Consanguineous marriage (F2) was noted in one of the 10 families in our cohort (Figure 4). After variant calling and data filtering, we identified 15 compound variants and two homozygous variants in the 12 patients with *BBS2* (F1–F5), *BBS4* (F6), *BBS7* (F7–F8), and *BBS9* (F9–F10) gene (Table 3), including five splice variations (c.263 + 2delT, c.534 + 1G > T, c.1198 + 1G > A, c.2059 + 1G > C, and c.2059 + 1G > T), three frameshift variations c.72delT(p.L25Cfs^∗^16), c.563delT(p.I188Tfs^∗^13), and c.1002delT(p.N335Ifs^∗^47), four nonsense variations c.31C > T(p.Q11^∗^), c.445C > T(p.R149^∗^), c.1015C > T(p.R339^∗^), and c.1395T > A(p.Y465^∗^), four missense variations c.79A > C(p.T27P), c.728G > A(p.C243Y), c.932G > A(p.G311D), and c.944G > A(p.R315Q), one synonymous variation c.1278A > G(p.E426E). These variants were confirmed as perfectly co-segregated with the disease within the families in which they occurred (Figure 4) based on a recessive pattern of inheritance as ascertained by Sanger sequencing. Among the identified variants, c.563delT(p.I188Tfs^∗^13), c.944G > A(p.R315Q), and c.1015C > T(p.R339^∗^) in the *BBS2* gene (Katsanis et al., 2001; Xing et al., 2014; Shaheen et al., 2016), c.728G > A(p.C243Y) and c.1002delT(p.N335Ifs^∗^47) in the *BBS7* gene (Chen et al., 2013; Wang et al., 2013) have been reported to be related to RP or BBS, while the rest of the 12 variants in the four genes were never been reported in ClinVar or HGMD Professional, so they were considered novel (*BBS2*, 41.67%; *BBS4*, 16.67%; *BBS7*, 8.33%; and *BBS9*, 33.33%). Moreover, according to the ACMG guidelines, except for c.79A > C(p.T27P) and c.1278A > G(p.E426E) which were classified as uncertain significance, we classify the other 15 variants as pathogenic. Furthermore, in this study, the novel heterozygous variation c.534 + 1G > T in *BBS2* was detected in two families (F3 and F5), and, among the pathogenic *BBS* genes in the 10 probands, *BBS2* was the most common.

**TABLE 3:** Identified variants in patients with BBS.

Patient	Gene*	Exon/Intron	Nucleotide change	Protein change	Hom/Het	PolyPhen-2	PROVEAN	SIFT	Mutation Taster	HSF	gnomAD	Rs	Report	ACMG
F1-II1	BBS2	Intron16	c.2059 + 1G > C		het	–	–	–	DC	MPADS	–	–	Novel	P (PVS1, PM2, PM3, PP1)
		Exon5	c.563delT	p.I188Tfs*13	het	–	–	–	DC	PADS	0.00001083	rs1367927635	Xing et al., 2014	P (PVS1, PS1, PM2, PM3)
F2-II:1, F2-II:2	BBS2	Exon1	c.79A > C	p.T27P	hom	benign	Neutral	Tolerated	Polymorphism	PADS	–	–	Novel	US (PM1, PM2, PP1, BP4)
F3-II:1, F3-II:2	BBS2	Intron4	c.534 + 1G > T		het	–	–	–	DC	MPADS	0.000101	rs773862084	Novel	P (PVS1, PM2, PP1)
		Exon11	c.1278A > G	p.E426E	het	–	Neutral	Tolerated	DC	PADS	–	–	Novel	US (PM2, PM3, PP1, BP4)
F4-II:1	BBS2	Exon9	c.944G > A	p.R315Q	het	PRD	Deleterious	Damaging	DC	PAAS	–	rs544773389	Katsanis et al., 2001	P (PS1, PM1, PM2, PM3, PP3)
		Exon9	c.1015C > T	p.R339*	het	–	–	–	DC	No impact	–	rs193922710	Shaheen et al., 2016	P (PVS1, PS1)
F5-II:1	BBS2	Intron16	c.2059 + 1G > T		het	–	–	–	DC	MPADS	–	–	Novel	P (PVS1, PM2, PM3, PP1)
		Intron4	c.534 + 1G > T		het	–	–	–	DC	MPADS	0.000101	rs773862084	Novel	P (PVS1, PM2, PP1)
F6-II:1	BBS4	Exon12	c.932G > A	p.G311D	het	PRD	Deleterious	Damaging	DC	No impact	–	–	Novel	P (PM1, PM2, PM3, PP1, PP3)
		Exon2	c.31C > T	p.Q11*	het	–	–	–	DC	PADS	–	–	Novel	P (PVS1, PM2, PP1)
F7-II:1	BBS7	Exon10	c.1002delT	p.N335Ifs*47	hom	–	–	–	DC	No impact	–	–	Chen et al., 2013	P (PVS1, PS1)
F8-II:1	BBS7	Exon8	c.728G > A	p.C243Y	het	PRD	Deleterios	Damaging	DC	No impact	0.00003974	rs727503821	Wang et al., 2013	P (PS1, PM2, PM3, PP1, PP3)
		Exon14	c.1395T > A	p.Y465*	het	–	–	–	DC	PADS	–	–	Novel	P (PVS1, PM2, PP1)
F9-II:1	BBS9	Exon6	c.445C > T	p.R149*	het	–	–	–	DC	PAAS	0.00001224	rs781174906	Novel	P (PVS1, PM2, PP1)
		Intron3	c.263 + 2delT		het	–	–	–	DC	MPADS	–	–	Novel	P (PVS1, PM2, PM3, PP1)
F10-II:1	BBS9	Exon2	c.72delT	p.L25Cfs*16	het	–	–	–	DC	No impact	–	–	Novel	P (PVS1, PM2, PM3, PP1)
		Intron10	c.1198 + 1G > A		het	–	–	–	DC	MPADS	–	–	Novel	P (PVS1, PM2, PM3)

DC, disease causing; LP, likely pathogenic; MPADS, most probably affecting donor splicing; P, pathogenic; PAAS, potential alteration of acceptor splice site; PADS, potential alteration of donor splice site; POD, possibly damaging; PRD, probable damaging; –, not available; US, uncertain significance. *The transcripts of the four genes we used in this study for sequencing and refer were *BBS2* (NM_031885.3), *BBS4* (NM_033028.4), *BBS7* (NM_176824.2), and *BBS9* (NM_198428.2).

### Analysis of BBS Related Literatures

We performed a literature analysis of the BBS related literatures on both Chinese and foreigner patients (number of cases ≥ 7), focusing on the ocular characteristics shown in Table 4. The first report of a BBS case in the Chinese literature was in 1954. So far, more than 100 BBS patients distributed among 17 provinces in China have been reported (Wei et al., 1998), Among these cases, the initial eye symptoms were most of night blindness, vision decline, so as the foreign reports (Deveault et al., 2011; Berezovsky et al., 2012; Castro-Sánchez et al., 2015; Scheidecker et al., 2015; Esposito et al., 2017). The age at diagnostic exam ranged from 1 to 65 years, and most were later the actual age of onset. The consanguineous mating rate (10% in our study) ranged between 35 and 39% in these previous studies. Among these reports (Table 4), not all BBS cases met the criteria of four main characteristics or three main characteristics plus two secondary characteristics (Hulleman et al., 2016). However, the high penetrance of RP seemed to be universal among all the BBS patients. Moreover, compared with the other five main clinical manifestations, the proportion of renal abnormalities was found to be relatively low (Wei et al., 1998). Variations in *BBS1*, *BBS2*, and *BBS10* are the most common disease-causing genes among BBS patients in abroad (responsible for 50% of all cases). Thus far, our report, together with other local Chinese patient reports, suggests that the *BBS2* (20%) and *BBS7* (14.29%) genes are hot spot genes among Chinese BBS patients.

**TABLE 4:** Clinical manifestations and disease-causing genes of patients with BBS from previous reports^#^.

							Abnormal			
	
Number (male/female)	Age at diagnostic exam	Initial eye symptoms	RP	Polydactyly	Obesity	Intellectual disability	Renal	Gonad	Disease-causing gene (Top 3)	Report
**Cases outside China**										
83 (42/41)	mean age of 19 y, age range 50–64 y	Most are vision decline, nystagmus	100%	97%	92%	61%	53%	59%	BBS10 (*N* = 23), BBS1/BBS2 (*N* = 16), BBS12 (*N* = 12)	Deveault et al. (2011)
23 (15/8)	mean age of 15y, age range of 6∼36 y	Most are night blindness, vision decline, vision field defect	100%	78%	91%	78%	30%	–	–	Berezovsky et al. (2012)
62 (30/32)	mean age of 21 y, age range 16–56 y	–	100%	–	–	–	–		BBS1 (*N* = 35), BBS2 (*N* = 6), BBS10 (*N* = 5)	Denniston et al. (2014)
52 (28/24)	mean age 6y	Most are night blindness, vision decline, vision field defect	100%	79%	88%	56%	23%	33%	BBS1(*N* = 33), BBS10 (*N* = 9), BBS12 (*N* = 8)	Castro-Sánchez et al. (2015)
7 (6/1)	mean age of 37 y, age range of 25∼54 y	Most are vision decline	100%	100%	83%	42%	14%	42%	BBS10 (*N* = 3), BBS1/BBS5/BBS6/BBS12 (*N* = 1)	Scheidecker et al. (2015)
12	mean age of 25y, age range of 9–65 y	vision decline	100%	–	–	–	75%	–	BBS1(*N* = 6), BBS1(*N* = 4), BBS2 (*N* = 2)	Esposito et al. (2017)
**Domestic cases in China**										
*65 (45/20)	1∼37 y	–	72%	75%	89%	90%	10%	–	–	Wei et al. (1998)
2 (2/0)	32 y, 37 y	–	Yes	Yes	Yes	Yes	Yes	Yes	BBS7 (*N* = 2)	Yang et al. (2008)
6 (4/2)	–	–	Yes	Yes	Yes	Yes	–	16%	BBS2/BBS3 (*N* = 2), BBS6/BBS13 (*N* = 1)	Xing et al. (2014)
1 (0/1)	4 y	Night blindness, Vision decline	Yes	Yes	Yes	Yes	–	No	BBS4 (*N* = 1)	Li et al. (2014)
2 (1/1)	6 y	–	Yes	Yes	N0	Yes	Yes	Yes	BBS6 (*N* = 2)	Qi et al. (2017)
3 (1/2)	35 y, 37 y, 39 y	–	Yes	Yes	Yes	Yes	Yes	1M, the rest one are unknown	BBS7(*N* = 3)	Shen et al. (2019)
1 (1/0)	4 y	Night blindness, Vision decline, Vision field defect	Yes	Yes	Yes	N0	–	–	BBS2 (*N* = 1)	Dan et al. (2020)
5	–	–	20%	60%	80%	60%	–	–	BBS2 (*N* = 2), TM1M67/BBS12 (*N* = 1)	Tang et al. (2020)
7 (6/1)	Mean age of 12 y, age range of 5∼22 y	Night blindness, Vision field defect	Yes	Yes	Yes	Yes	1M, the rest are unknown	–	BBS2/BBS10 (*N* = 2)	Tao et al. (2020)

N, number; –, not available. ^#^Study aboard: number of patients ≥ 7, study in China: no limitation of number of patients. *This letter summarizes all cases of BBS reported in China since 1954.

## Discussion

The detailed clinical and genetic characteristics of a cohort of 12 affected subjects from 10 Chinese families with BBS are illustrated in this study. To our knowledge, this is largest cohort of BBS patients by diagnosis using comprehensive clinical and genetic analysis to date in China. Although BBS has similar features and molecular overlap with Usher syndrome, Alström syndrome (OMIM 203800), McKusick-Kauffman (OMIM 604896) syndrome, Joubert syndrome (OMIM 213300, 608091, 608629, 609583, 610688, 611560, 61229, 612285, and 300804) or Meckel Gruber syndrome (OMIM 249000, 603294, 607361, and 611134), they are considered distinct clinical diseases, such as Alström syndrome (Pereiro et al., 2010) and Usher syndrome (Tsang et al., 2018) no polydactyly, or hypogonadism. Advanced gene sequencing technology has played an important role in helping distinguish between these similar genetic diseases. Although, *ALMS1* gene which belong to Alström syndrome was not tested genetically specially by the patients (F1-II:1, F3-II:1, and F3-II:2) which have hearing disturbances, the disease-causing gene of BBS we identified was co-segregated with the family members and phenotype. The BBS patients in our study did not all fully meet the clinical diagnosis criteria for BBS, especially with the low proportion of renal abnormalities. Wei et al. (1998) reviewed 65 Chinese cases published from 1956 to 1998, finding that renal abnormalities of patients accounted for only 10.6%, and only 44.6% of patients had five main features. Furthermore, the ratio of renal abnormalities in cases reported abroad has been very different (Table 4) (Imhoff et al., 2011), as the phenotypes of BBS cases in different regions have differed, including the unexpected high frequency of tooth anomalies in our cohort, which further confirmed the high heterogeneity phenotype of BBS patients.

In this study, we focus on the ocular characteristics of BBS patients and explore the similarities and differences between the cohort we reported and previous cases reported in China and abroad. It is always challenging for doctors to access the complete information regarding the development of the retina of BBS patients, since patients present with visual symptoms at a young age and their visit to an ocular department usually occurs later than their actual onset. Impaired vision and night blindness have been found to be the most common initial eye symptom in patients worldwide. In our study, 12 patients exhibited low vision and night blindness which was independent age and gender. Weihbrecht et al. (2017) reported more than 90% of individuals with BBS displayed rod-cone dystrophy with general early macular involvement. They also observed the retina morphology by fundus, FAF, and OCT, and accessed the retinal function via ffERG and mfERG. We found that the visual field could not be measured accurately in these BBS patients because the vision of these patients was considered too poor to perform the kinetic visual field testing. And in the objective vision function assessment via visual electrophysiology, the ffERG wave was mostly unrecordable. Scheidecker et al. (2015) reported macular atrophy in all BBS patients, and severely reduced and delayed ERG-light-adapted responses were found in most BBS patients. In general, FVEP testing can appraise the residual visual function of BBS patients, and FVEP changes seem to be consistent with BCVA impairment, suggesting the feasibility of using FVEP to assess residual visual function in patients with rod-cone dystrophy without detection of FERG response.

Researcher made effort to find the genotype-phenotype correlations in BBS families (Table 4) (Deveault et al., 2011; Daniels et al., 2012; Niederlova et al., 2019), but actually, it is high diversity among affected patients that existed the same genotype from one family (F2 and F3 in our study). In our study, we found that the clinical manifestations of the different genes did not differ significantly, and five patients with the same disease-causing gene in *BBS2* had completely different fundus morphological and functional changes. This indicated that the *BBS* genes and phenotypes are highly heterogeneous, which is consistent with previous findings (Table 4). In our study, we identified four pathogenic *BBS* genes (*BBS2*, *BBS4*, *BBS7*, and *BBS9*). Of those, *BBS2* was the most common in our cohort. We also identified a novel *BBS2* heterozygous variant c.534 + 1G > T in two families. Due to the small number of cases included in this study, it was not clear whether this is a hot spot variant of *BBS* in the Chinese population. However, the gene and its regional distributions, and even the distributions of hot spot variants have differed among studies (Mykytyn et al., 2002; Katsanis, 2004; Stoetzel et al., 2006; Ajmal et al., 2013). Yet variation in *BBS1*, *BBS2*, and *BBS10* are the most common disease-causing genes among BBS patients abroad (responsible for 50% of all cases).

Overall, our study had some limitations. First, the number of enrolled patients was small. Second, we did not perform a longitudinal study regarding visual impairment. The changes in morphology and function of the retina should be followed up and correlated with different genes. Therefore, future studies with a larger sample size of BBS patients with BBS variants should be conducted using longitudinal clinical assessments and genotype-phenotype correlation analyses, not only in retina but also nephrology and endocrinology investigations and others to find some late syndromatic symptoms and signs (follow up by ophthalmologist, nephrologist and internist).

## Conclusion

In our study, it demonstrated heterogeneous visual function and retinal morphology changes in these BBS patients. Our study expands the variation spectrum of *BBS*, and indicates that a combination of ffERG and FVEP may be helpful in evaluating the residual bioelectric activity of retina and visual pathway. To our knowledge, this is the largest cohort study with BBS patients in the Chinese population until now, we may conduct a large-scale screening BBS clinical cohort study among RP patients in China in the future.

## Data Availability Statement

The original contributions presented in the study are included in the article/Supplementary Material, further inquiries can be directed to the corresponding author/s.

## Ethics Statement

The studies involving human participants were reviewed and approved by Ethics Review Board of Southwest Hospital, Army Medical University (Third Military Medical University) (Chongqing, China, KY2020096). Written informed consent was obtained from the individual(s), and minor(s)’ legal guardian/next of kin, for the publication of any potentially identifiable images or data included in this article.

## Author Contributions

XM collected cases, evaluated clinical characteristics, molecular diagnosis, and wrote the manuscript. YL made the figures and tables, and management data. GW helped in data evaluating. JR and XY collected and organized the data. SL collected cases, and wrote and revised the manuscript.

## Conflict of Interest

The authors declare that the research was conducted in the absence of any commercial or financial relationships that could be construed as a potential conflict of interest.

## Supplementary Material

Supplementary Figure 1 The mfERG recordings in patients with BBS. The mfERG waves were unrecordable in all patients accessed.

Supplementary Figure 2 PVEP recordings in patients with BBS. The PVEP results showed a severely decreased amplitude and moderate delayed peak time in the P100 wave of BBS patient F6-II:1, while the other patients had unrecordable PVEP due to nystagmus and fixation loss.

Supplementary Figure 3 Sanger sequencing of disease-causing variants were identified in BBS patients.

Supplementary Table 1 131 inherited retinal disease genes analyzed by targeted NGS diagnostic testing.
