# Supplementary figures and images for: Ocular Characteristics of Patients With Bardet–Biedl Syndrome Caused by Pathogenic BBS Gene Variation in a Chinese Cohort
Source: Front Cell Dev Biol. 2021 Mar 11;9:635216. doi: 10.3389/fcell.2021.635216 (PMC7991091; doi:10.3389/fcell.2021.635216)

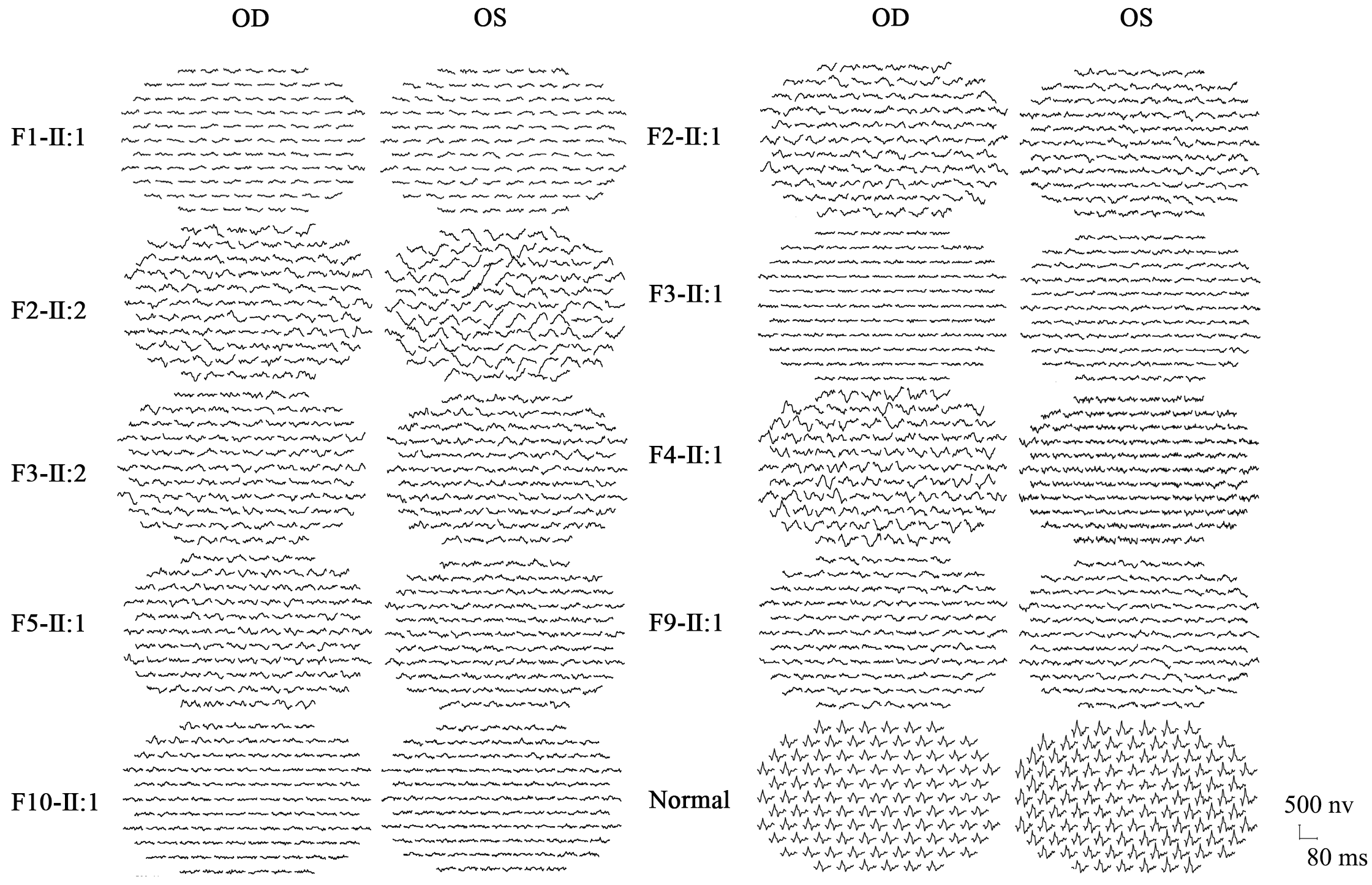

Supplement: Supplementary Figure 1 — The mfERG recordings in patients with BBS. The mfERG waves were unrecordable in all patients accessed. [file Image_1.TIF]

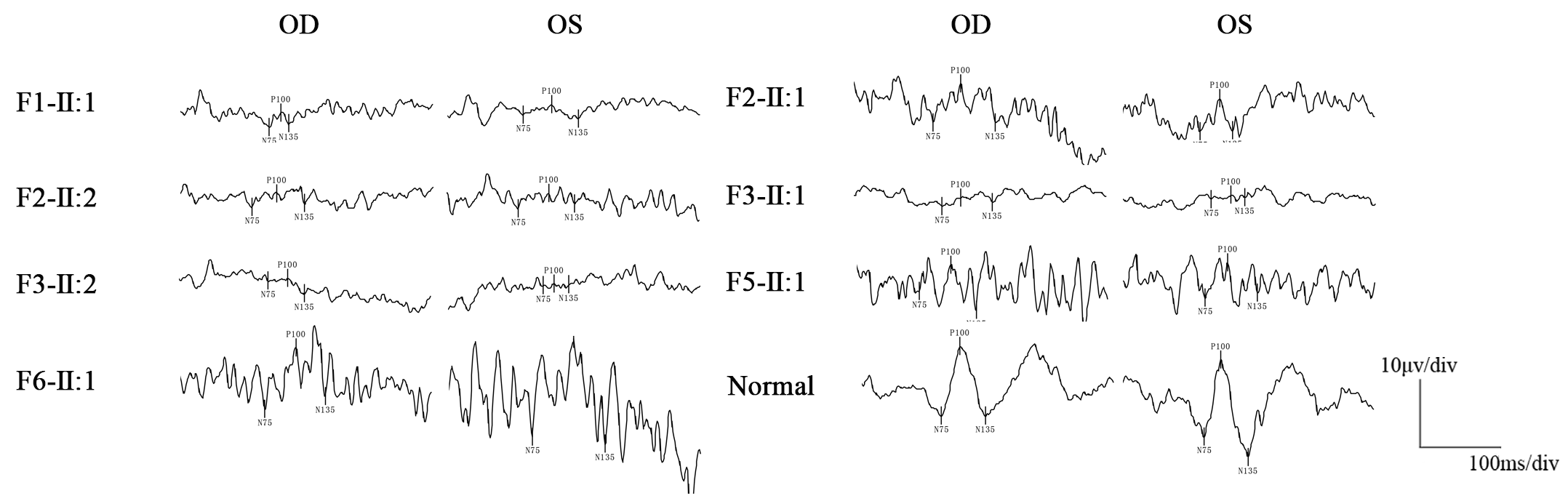

Supplement: Supplementary Figure 2 — PVEP recordings in patients with BBS. The PVEP results showed a severely decreased amplitude and moderate delayed peak time in the P100 wave of BBS patient F6-II:1, while the other patients had unrecordable PVEP due to nystagmus and fixation loss. [file Image_2.TIF]

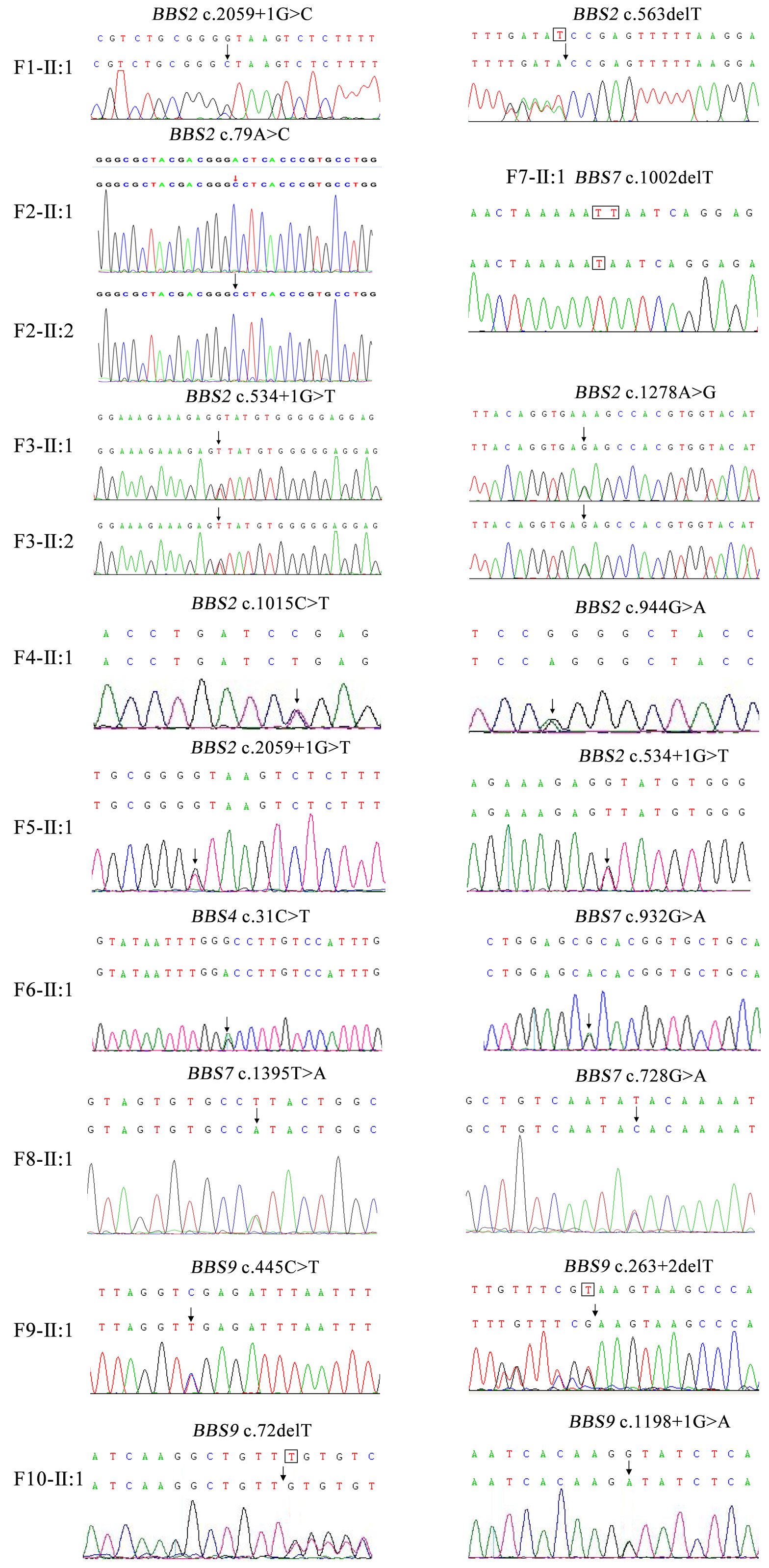

Supplement: Supplementary Figure 3 — Sanger sequencing of disease-causing variants were identified in BBS patients. [file Image_3.TIF]
