# Supplementary material for: Ocular Characteristics of Patients With Bardet–Biedl Syndrome Caused by Pathogenic BBS Gene Variation in a Chinese Cohort
Source: Front Cell Dev Biol. 2021 Mar 11;9:635216. doi: 10.3389/fcell.2021.635216 (PMC7991091; doi:10.3389/fcell.2021.635216)
Supplement: Supplementary Table 1 — 131 inherited retinal disease genes analyzed by targeted NGS diagnostic testing. [file Table_1.DOCX]

| *ABCA4* | *BEST1* | *DHX38* | *IMPG1* | *NR2E3* | *RDH12* | *SNRNP200* |
| --- | --- | --- | --- | --- | --- | --- |
| *ABCB6* | *C1QTNF5* | *DRAM2* | *IMPG2* | *NRL* | *RDH5* | *SPATA7* |
| *ABCC6* | *C2orf71* | *EFEMP1* | *KCNJ13* | *OFD1* | *REEP6* | *SPP2* |
| *ADIPOR1* | *C5orf42* | *ELOVL4* | *KCNV2* | *OTX2* | *RGR* | *TIMP3* |
| *AGBL5* | *C8orf37* | *EMC1* | *KIAA1549* | *PDE6A* | *RHO* | *TMEM67* |
| *AHR* | *CA4* | *EYS* | *KIZ* | *PDE6B* | *RLBP1* | *TOPORS* |
| *ARHGEF18* | *CCDC28B* | *FAM161A* | *KLHL7* | *PDE6G* | *ROM1* | *TRNT1* |
| *ARL2BP* | *CEP290* | *FSCN2* | *LCA5* | *POMGNT1* | *RP1* | *TTC8* |
| *ARL3* | *CERKL* | *GNAT1* | *LRAT* | *PRCD* | *RP1L1* | *TTC8* |
| *ARL6* | *CFH* | *GPR125* | *LZTFL1* | *PRDM13* | *RP2* | *TULP1* |
| *BBIP1* | *CLCC1* | *GUCA1B* | *MAK* | *PROM1* | *RP9* | *USH1C* |
| *BBS1* | *CLRN1* | *HGSNAT* | *MERTK* | *PRPF3* | *RPE65* | *USH1G* |
| *BBS10* | *CNGA1* | *HK1* | *MFSD8* | *PRPF31* | *RPGR* | *USH2A* |
| *BBS12* | *CNGB1* | *HMCN1* | *MKKS* | *PRPF4* | *RPGRIP1* | *VCAN* |
| *BBS2* | *CRB1* | *IDH3B* | *MKS1* | *PRPF6* | *RPGRIP1L* | *WDPCP* |
| *BBS4* | *CRX* | *IFT140* | *MVK* | *PRPF8* | *SAG* | *ZNF408* |
| *BBS5* | *CTNNA1* | *IFT172* | *MYO7A* | *PRPH2* | *SAMD11* | *ZNF513* |
| *BBS7* | *CYP4V2* | *IFT27* | *NEK2* | *PTHB1* | *SEMA4A* |  |
| *BBS9* | *DHDDS* | *IMPDH1* | *NEUROD1* | *RBP3* | *SLC7A14* |  |

**Supplementary Table1. 131 inherited retinal disease genes analyzed by targeted NGS diagnostic testing.**
